# Supplementary material for: Platelet-Derived miR-126-3p Directly Targets AKT2 and Exerts Anti-Tumor Effects in Breast Cancer Cells: Further Insights in Platelet-Cancer Interplay
Source: Int J Mol Sci. 2022 May 13;23(10):5484. doi: 10.3390/ijms23105484 (PMC9141257; doi:10.3390/ijms23105484)
Supplement: Supplementary file 1 [file ijms-23-05484-s001.zip › ijms-1717273-supplementary.pdf]

International Journal of
Molecular Sciences

MDPI

Supplementary Materials

(a)

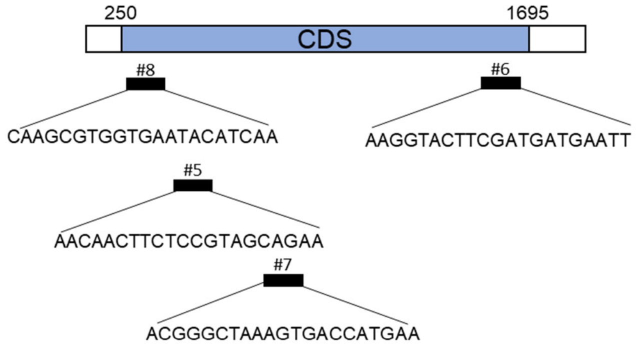

(b)

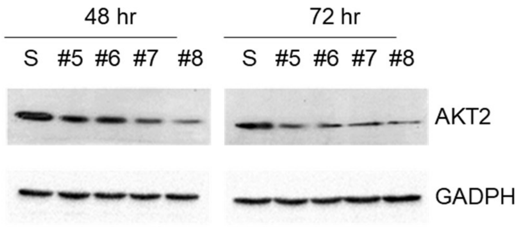

(c)

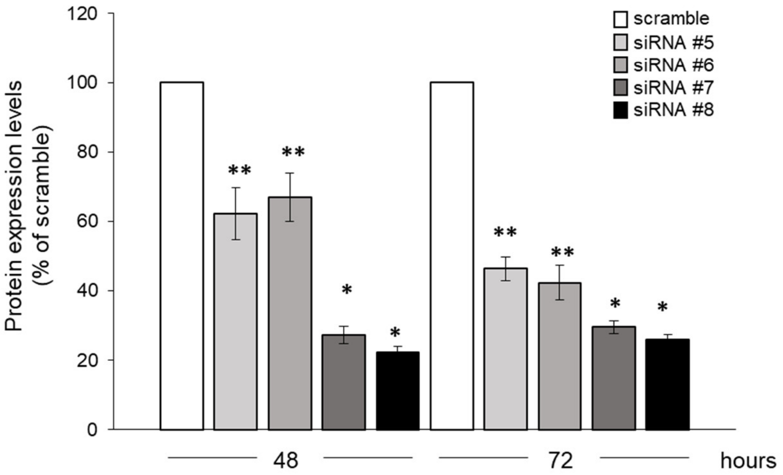

Figure S1. Setting of AKT2 silencing conditions in BT549 cells. (**a**) Sequences of siRNA oligonucleotides designed to target different regions of AKT2. The coding sequence (CDS) of AKT2 is also displayed. (**b**) Western blot analysis of AKT2 expression in BT549 cells transfected with each AKT2 siRNA or with scramble oligo for 48 and 72 hours. Blots are representative of five independent experiments. GAPDH was used as loading control. (**c**) Histograms show densitometric analysis of AKT2 expression levels compared to scramble-transfected cells, arbitrarily set to 100%. Values are reported as mean ± SEM. *: p <0.01 and ** p <0.05 versus scramble.
